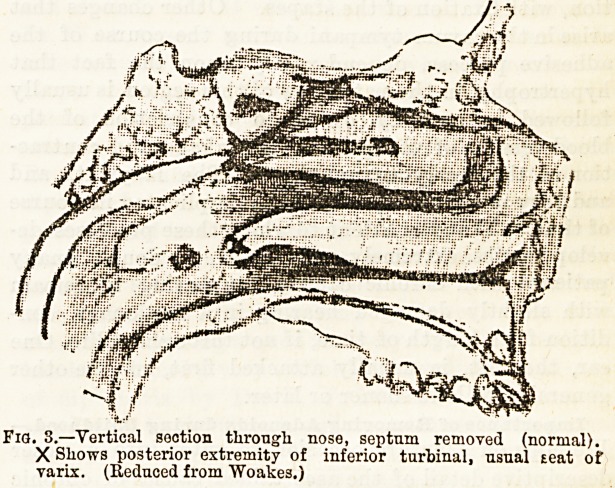# Chronic Catarrh of the Middle Ear

**Published:** 1894-08-04

**Authors:** L. H. Pegler


### MEDICAL PROGRESS AND HOSPITAL CLINICS.

[*The Editor will be glad to receive offers of co-operation and contributions from members of the profession. All letters should be addressed to* THE EDITOR, THE LODGE, PORCHESTER SQUARE, LONDON, W.]

### CHRONIC CATARRH OF THE MIDDLE EAR.—*Continued.*

By L. H. PEGLER, M.D., M.R.C.S.

**Chronic forms of Middle Ear Catarrh.—Etiology.**—From the recent mucous catarrh of acute rhinitis, we pass to a large class of cases in which the middle ear catarrh is an accompanying symptom of some less evanescent

FIG. 1.—"Roof" adenoids as seen by posterior rhinoscopy. (From Woakes' "Post-Nasal Catarrh")

obstruction in the nose or nasopharynx. In the first instance there is probably a similar commencement, but with more permanent causes in operation there is less tendency to spontaneous recovery, and greater proneness for the aural complications to become chronic.

**Tonsillar, Turbinal, and Adenoid Hypertrophies.**—Amongst the more important of these etiological factors (excluding the specific fevers, syphilis, and many others which we cannot enumerate) are enlarged tonsils and adenoid hypertrophies; these two conditions in children are so frequently associated, that the probable existence of the latter should always be kept in mind when treating the former (Lennox Browne). Adenoid growths often completely fill the space behind the nose; not only obstructing the posterior nares, but the Eustachian orifices as well, the cushions or lips of which are often themselves the site of lymphoid overgrowth (Fig. 1). After adolescence, other pathological conditions

FIG. 2.—Post-turbinal varix, as seen by rhinoscopic mirror. Moriform growth removed, (From Woakes' "Post-Nasal Catarrh," 1884.)

are apt to spring up and create nasal and naso-pharyngeal obstruction, namely, polypi, mucous or fibrous, crowding the meati, and often, too, the post-nasal space; polypoid hyperthrophies of the mucosa covering the middle turbinated body, and generally mistaken for polypi; together with various other growths and septal deformities which occasion nasal stenosis. Special mention must, however, be made of the changes that are liable to overtake the inferior turbinated body. The erectile tumefactions to which it is subject, the polypoid neoplasms and hypertrophies that frequently project from its anterior and posterior extremities (Fig. 2), as well as along its continuity, and occlude the inferior, or respiratory, meatus of the nose, bring the lower turbinal into still closer relationship with the etiology of chronic aural and Eustachian catarrh. It will be readily understood that hypertrophies of this body, when developed posteriorly, must encroach very closely upon the mouth of the Eustachian tube, and interfere seriously with its drainage.

**Paresis of Soft Palate.**—Another point is that, when resting upon the upper or posterior surface of the soft palate, these posterior or moriform enlargements (the "turbinal varix" of Wingrave) hamper the functions of the levator and tensor palati (dilator tubæ) muscles which ventilate the tube and middle ear during the acts of speaking and swallowing, and in this way render the palate paretic (Fig. 3).

FIG. 3.—Vertical section through nose, septum removed (normal). X Shows posterior extremity of inferior turbinal, usual seat of varix. (Reduced from Woakes.)

Other impediments to the proper action of the palate fall into the same category as a cause of Eustachian catarrh. Enlarged tonsils have already been mentioned, and to these may be added post-diphtheritic paralysis, relaxed and hypertrophied uvula, also thickenings of the folds of mucous membrane covering the salpingo-pharyngeal muscles—the pharyngeal bands. When also the naso-pharynx is full of adenoid growths, or of polypi, paresis of the palate is almost certain to result.

These and many other allied deviations from the normal are amenable to surgical treatment, and the associated aural symptoms will disappear, if such treatment be intelligently carried out, and not unduly deferred. How long a period may be suffered to elapse before structural changes take place in the tympanum, which will render the hardness of hearing permanent, must depend upon individual peculiarities. The exciting cause remaining, there is certainly but little tendency to spontaneous amelioration (unless of an extremely evanescent character), however much truth there may be in the assertion that the cell infiltration of exudative catarrhs of the tympanic mucous membrane tends to disintegrate and resolve.

**Clinical History of Middle Ear Catarrh.**—The clinical history of such cases is probably somewhat as follows: A temporary and curable interference with audition is induced in the first instance by blocking of the Eustachian tube, indrawing of the membrana tympani and clogging of the ossicular chain by mucous secretion: the less fluid and more tenacious the secretion, the more complete being the interference with the mechanism of the ossicles. The removal of the exudate naturally, or by treatment, failing to take place, and in unfavourable cases, in spite perhaps of either, the risk of complication by selerosis becomes superadded at this juncture. In other words, simultaneously with or subsequent to the absorption of the secretions, a hypertrophy and proliferation of the epithelial investments of the tympanic chambers, membrane, and ossicles may now take place, rendering the hindrance to the performance of their functions permanent. Thus we get thickening, in addition to indrawing of the membrana tympani, and often the formation of chalky opacities within its layers. More serious still is the liability to rigidity and ankylosis of the ossicular joints, from a similar calcareous deposition, with fixation of the stapes. Other changes that arise in the cavum tympani during the course of the adhesive process, depend partly upon the fact that hypertrophy, in the pathology of this region, is usually followed by atrophy, owing to constriction of the blood-vessels of the parts by shrinkage and contraction of the newly-formed tissue. The labyrinth and auditory nerve are apt to become implicated in course of time. That the extent to which these processes develop varies extremely, admits of no doubt, many patients with chronic catarrhs appearing to remain with slightly damaged hearing in a stationary condition for a length of time, if not throughout life. One ear, the left, is usually attacked first, but the other generally follows sooner or later.

**Importance of Removing Adenoids during Childhood.**—We hope on a future occasion to enter into a closer descriptive detail of the ascertained causes of chronic mucous catarrh, with especial reference to their diagnosis and treatment; in the meantime we may state that of those that have been enumerated in this paper, it is probable that adenoid hypertrophies of the naso-pharynx, and enlarged tonsils, in childhood, are the most frequent and potential in laying the foundation of impairment of hearing. This inference is irresistible when we contemplate how many children are brought up for treatment already deaf with dry or discharging ears, who are the subjects of these conditions, especially adenoids, and how remarkably both deafness and discharge will clear up, in the large majority of cases, when they are removed. Hence the extreme importance of an acquaintance with the diagnosis of these growths on the part of the family medical attendant. It is to be hoped that the increased attention which is already being paid to the discovery and treatment of these and other sources of nasal obstruction in the young will render deafness a less common infirmity in the generations that are to come. It may be true that large numbers of young people ask advice for impairment of hearing, in whom nothing very evident reveals itself, calling for surgical ablation; but a comparatively insignificant lymphoid hyperplasia in the naso-pharynx tending to shrink and disappear at puberty, can, it must be remembered, set up a disastrous secondary catarrh within the narrow confines of the tympanic cavity, where permanent changes in the hearing apparatus gradually manifest themselves after the primary etiological element in the case has possibly dwindled and become inconspicuous. In a fair proportion of these patients, nevertheless, one is able to discover by taking pains, sufficient traces of past abnormal conditions to account for the otitis media which eventually resulted in deafness. That many people who consult us on account of nasal and pharyngeal obstructions evince a present immuuity from middle ear implication is a fact which we must account for hypothetically; still, we are justified in assuming that the same risk is always pending, and that continued neglect might incur an invasion of the ear in course of time.

**Rhinitis Sicca and Middle Ear Catarrh.**—We have spoken of certain hypertrophic states of the nasal mucous membrane occasioning obstruction to respiration, as a cause of catarrh of the middle ear; it must also be added that there is a frequent association of chronic deafness with those diseases of the nose, of which one of the special characters is dryness with inspissation, or crusting of the secretions.

For our present purpose, it will be sufficient to mention two, as most frequently illustrating this point, viz., rhinitis sicca, so commonly seen in gouty and alcoholic subjects and anæmic women; andrhini tis atrophica, popularly known as ozœna, from the fœtor exhaled in that disease. When deafness is a concomitant of dry rhinitis, it is usually very intractable, the ear suffering secondarily like the pharynx and larynx, for the voice organs are commonly implicated as well. To what degree the atrophic process, which is so apparent on the posterior wall of the pharynx, extends itself along the Eustachian tube, it is difficult to say; but we surmise from the present limited state of our information, that chronic otitis media, when found in this connection, is most probably of the so-called "dry," "atrophic," or sclerotic type, respecting which we must, in conclusion, make a few further observations.

**Sclerosis.**—This form of chronic middle ear catarrh without apparent inflammatory exudation, or mucous secretion, was formerly, by many authors, and still is by some, classed separately with the title of *plastic otitis media* (Gruber), or *sclerosis*; but the tendency with otologists, more recently with reference to this somewhat obscure subject, is to regard the sclerosing process as too prone to be developed upon chronic exudative catarrh at almost any stage of its history for anything like a clear line to be drawn between the two; hence the distinction is not now considered to be of so much importance, at least by some advanced American writers, although Politzer and others appear to take a different view.

The super-addition just mentioned is especially liable to occur in people of a rheumatic, gouty, strumous or syphilitic diathesis. In typical idiopathic sclerosis, adhesive processes are pre-eminently characteristic and take the form of adventitious bands and fibres, which create all manner of displacements and dislocations in the relative position of tympanic membranes and ossicles to the walls and to each other. These changes set in early and insidiously, especially, it is believed, ankylosis of the stapes to the oval window, with consecutive extension of mischief to the labyrinth and auditory nerve, and to the air spaces in the mastoid bone. The Eustachian tube is usually spoken of as patulous, but it is equally probable that in later stages at least it may become completely stenosed. Hereditary predisposition is acknowledged by all writers to have a marked influence in the etiology of typical sclerosis, though the fact is variously accounted for; it is also frequently seen in conjunction with the neurotic temperament. Young women as well as elderly people are common subjects, and they complain at an early period of tinnitus; they also hear better when a noise is going on around. (Willis' paracusis). None of these symptoms, however, are peculiar to primary sclerosis; they are quite common as late developments in old-standing moist catarrhs, and, in fact, indicate the presence of pathological alterations that may overtake every kind of chronic middle ear disease, not excluding old perforations—namely, fixation of the ossicles and ankylosis of the stapes. The principal difference lies in the fact that here they set in idiopathically with insidious deafness, and without any apparent history of a previous catarrhal attack.

(*To be continued.*)

## Figures and Tables

**Fig. 1. f1:**
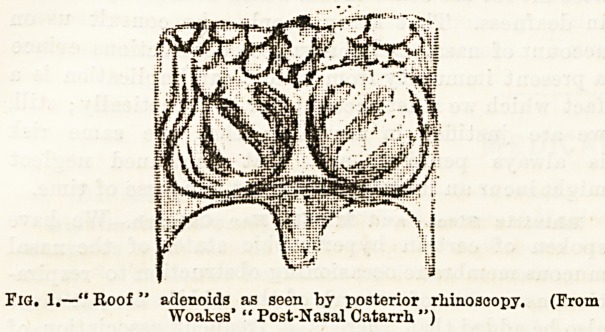


**Fig. 2. f2:**
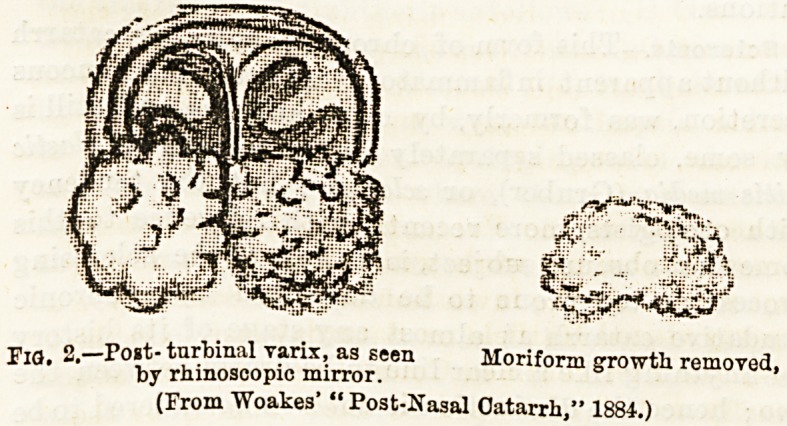


**Fig. 3. f3:**